# Divergent Primary Immune Responses Induced by Human Immunodeficiency Virus-1 gp120 and Hepatitis B Surface Antigen Determine Antibody Recall Responses

**DOI:** 10.1007/s12250-018-0074-6

**Published:** 2018-12-19

**Authors:** Li Yuan, Wen-Jiang Chen, Jia-Ye Wang, Yan Li, Dan Tian, Ming-Xia Wang, Hao-Tong Yu, Ying-Chu Xu, Di Li, Min Zhuang, Hong Ling

**Affiliations:** 1 Department of Microbiology, Harbin Medical University, Harbin, 150081 China; 2 Heilongjiang Provincial Key Laboratory of Infection and Immunity, Key Laboratory of Pathogen Biology, Harbin, 150081 China; 3 Wu Lien-Teh Institute, Harbin Medical University, Harbin, 150081 China; 4 Department of Immunology, Harbin Medical University, Harbin, 150081 China

**Keywords:** Human immunodeficiency virus type 1 envelope, Hepatitis B surface antigen (HBsAg), Immune memory, Primary immune response, Programmed death-1 (PD-1)

## Abstract

The development of a vaccine based on human immunodeficiency virus type 1 (HIV-1) envelope glycoprotein (Env) that elicits potent protective antibodies against infection has been challenging. Recently, we compared the antibody production patterns of HIV-1 Env gp120 and hepatitis B virus surface antigen (HBsAg) to provide insights into how we may improve the protective efficacy of Env-based immunogens. Our previous study showed that HIV Env and HBsAg display different mechanisms of antibody elicitation and that T cells facilitate the responses to repeated immunizations. Here, to elucidate the detailed roles of primary immunization in immune memory response formation and antibody production, we immunized C57BL/6 mice with each antigen and evaluated the development of T follicular helper (Tfh) cells, germinal centers, and the memory responses involved in prime and boost immunizations. We found that after prime immunization, compared with HBsAg, gp120 induced higher frequencies of Tfh cells and programmed death (PD)-1^+^ T cells, greater major histocompatibility complex II expression on B cells, comparable activated B cells, but weaker germinal center (GC) reactions and memory B cell responses in the draining lymph nodes, accompanied by slower antibody recall responses and poor immune memory responses. The above results suggested that more PD-1^+^ T cells arising in primary immunization may serve as major contributors to the slow antibody recall response elicited by HIV-1 Env.

**Electronic supplementary material:**

The online version of this article (10.1007/s12250-018-0074-6) contains supplementary material, which is available to authorized users.

## Introduction

Developing a prophylactic vaccine is the most efficient long-term solution to the global pandemic of human immunodeficiency virus type 1 (HIV-1). HIV-1 envelope glycoprotein (Env) is the principal target for vaccine development, aiming to induce antiviral humoral and cellular-mediated immunity. However, to date, we lack an ideal strategy for designing Env-based vaccines to elicit protective antibodies against HIV-1 infection.

In recent years, a collection of native-like Env trimers from different subtypes, based on the “SOSIP.664” design, have shown the ability to elicit potent neutralizing antibodies (NAbs) against autologous tier 2 strains but less potent NAbs against heterologous tier 2 strains (Sanders *et al.*^2015^; Klasse *et al.*^2016^; Wang *et al.*^2017^). Furthermore, the RV144 trial demonstrated 60% efficacy against infection at 6 months (Robb *et al.*^2012^) and 31% efficacy at 2 years in a community cohort (Rerks-Ngarm *et al.*^2009^). Partial protection using the native-like Env trimers in animal studies and the alternative Env immunogens in the RV144 clinical trial have shown promising results in Env-based vaccine design, although the concept of immunogen design is quite different. In addition, because information on the mechanisms of protection is still scarce, it is unclear how to further improve the efficacy of protection for these immunogens.

Hepatitis B virus (HBV) surface antigen (HBsAg) is the most effective vaccine antigen for reducing the global incidence of HBV (Romano *et al.*^2015^). Successfully vaccinated individuals usually show a rapid recall response to a booster several years after the primary vaccination or upon exposure to HBV (West and Calandra 1996). Two mechanisms are involved in the protection provided through HBsAg vaccination: neutralization of HBV by anti-HBsAg-specific antibodies and immune memory mediated by HBsAg-specific memory B (Bm) and memory T cells (West and Calandra 1996; Bauer and Jilg 2006; Brunskole Hummel *et al.*^2016^). Comparing the antibody production patterns of HBsAg and gp120 and exploring the possible mechanisms of antibody elicitation may provide insights into how to best improve the protective efficacy of Env-based immunogens. We previously found that, after several boosts, Env gp120 induced slower antibody recall responses, but redundant nonspecific IgG response compared with HBsAg (Yu *et al.*^2016^). The higher frequency of programmed death (PD)-1^hi^CD4^+^ T cells and T follicular helper (Tfh) cells elicited at an early time point following gp120 boost had limited the recall response (Yu *et al.*^2016^). Given that the quality and quantity of memory cells are set during the antigen-driven primary immune response (Sallusto *et al.*^2010^; Kurosaki *et al.*^2015^), it is important to explore the dynamics of immune activation of these cell populations and subsequent B cell responses in prime and boost processes, providing exact information on how we can intervene at prime immunization to improve immune protection.

In HIV-1-infected individuals, Tfh cells express high levels of the negative regulatory receptor PD-1. Interaction of PD-1 and its ligand PD-L1 on germinal center (GC) B cells can attenuate T cell receptor (TCR) signals, inhibit T-cell proliferation and activation, and inhibit production of the cytokine interleukin (IL)-21, which is crucial for B-cell survival and differentiation into plasma cells (Pillai 2013). The requirement for proper functional Tfh cells is indispensable for GC formation and for the differentiation of Bm and plasma cells (PCs) to generate high-affinity antigen-specific antibodies. However, the dynamics of the interactions among Tfh, GC B cells, and memory cells in the context of HIV-1 Env immunization are still unclear.

In addition to the need for designing more effective Env immunogens, it is also crucial to acquire an improved basic understanding of the unique primary immune responses against HIV-1 Env and its distinct influences on the different antibody recall and immune memory responses. Accordingly, in this study, we compared the primary immune responses after priming with HIV-1 gp120 and HBsAg. Our results may provide a direction for improving HIV Env immunogenicity by enhancing the recall response in the prime immunization phase.

## Materials and Methods

### Antigens and Adjuvant

The gp120 trimer proteins (gp120T), derived from HIV-1 subtype B’ (06044, EU131805) of a Chinese broad neutralizer, were constructed as previously described (Wang *et al.*^2011^; Yu *et al.*^2016^) and were expressed in Expi293F cells by transient transfection using an ExpiFectamine 293 Transfection Kit (Gibco, CA, USA) according to the manufacturer’s protocols. The cell culture supernatants were harvested at 4 days after transfection by centrifugation for 20 min at 8000 ×*g* and 4 °C and clarified through filtration with a 0.45-μm filter (Corning, NY, USA). The clarified culture supernatants were loaded onto lectin affinity columns (Vector Laboratories, Burlingame, CA, USA), and the bound gp120T proteins were eluted using 1 mol/L methyl α-d-mannopyranoside in phosphate-buffered saline (PBS, pH 7.4). The eluates were immediately dialysed in sterile PBS (pH 7.4) for buffer-exchange and then concentrated through an Amino Ultra Centrifugal Filter Unit with a 10-kDa cutoff (Millipore, Massachusetts, USA). The purified HBsAg proteins from infected donor plasma were purchased from GENIA Biotechnology Company (Beijing, China) and were shown to be well glycosylated (Wagatsuma *et al.*^2018^). Both gp120T and HBsAg were identified by native-polyacrylamide gel electrophoresis (PAGE) and western blotting in our previous study (Yu *et al.*^2016^). The proteins were formulated with an equal volume of the adjuvant AddaVax (InvivoGen, San Diego, CA, USA) and then used as immunogens. AddaVax is a squalene-based oil-in-water nano-emulsion with a formulation similar to that of MF59, comprising squalene oil and the nonionic surfactants Tween 80 and Span 85 (Fang and Hora 2000; Calabro *et al.*^2013^). AddaVax can also promote higher titers of protective Abs after re-exposure to the influenza vaccine (Hauck *et al.*^2018^; Souza *et al.*^2018^).

### Immunization and Specimen Harvesting

**Fig. 1:**
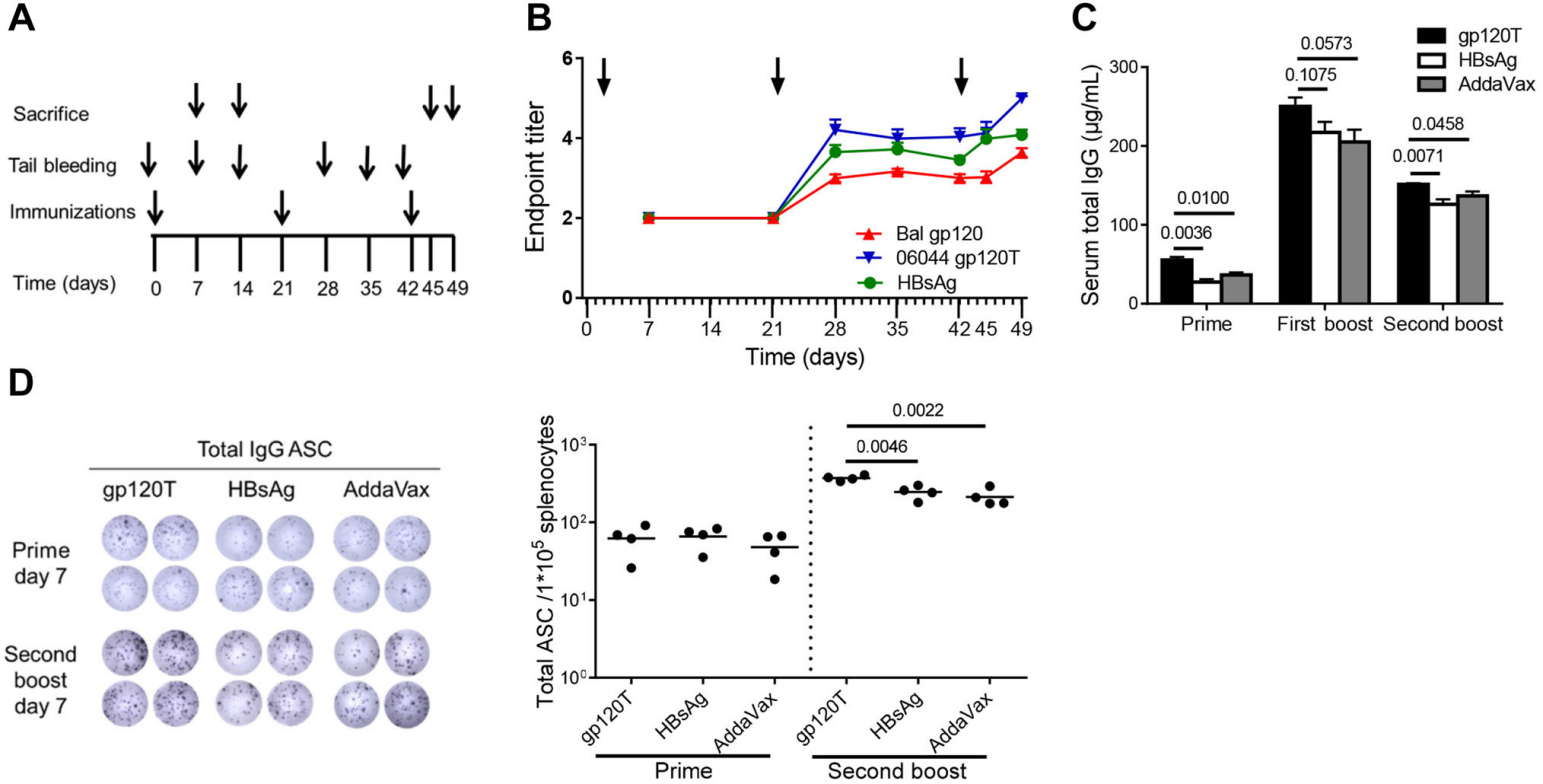
Humoral immune responses following immunization of C57BL/6 mice with gp120 and HBsAg (n = 4 per group). Mice were immunized subcutaneously with 15 μg wild-type trimeric gp120 (gp120T) or 2 μg HBsAg in AddaVax adjuvant formulation according to the immunization regimen (**A**). (**B**) The longitudinal plots of specific antibody responses to HIV-1 Bal gp120, HBsAg, and HIV-1 06044 gp120T were prepared after each immunization, and the endpoint titers are shown. Arrows indicate immunization. (**C**) The total IgG levels in immunized mouse serum on day 7 after each immunization were measured by quantitative ELISA. (**D**) Total specific antibody secreting cells (ASCs) on day 7 after prime (from 1 × 10^5^ splenocytes) and final boost (from 2 × 10^4^ splenocytes) were measured by B-cell ELISpot assays. Data are shown as means ± standard errors of the means (SEMs) from a single representative experiment of two independent experiments.

Female C57BL/6 mice (8–9 weeks old, 17–20 g) were immunized subcutaneously in the backs at weeks 0, 3, and 6 with 15 μg wild-type gp120T or 2 μg HBsAg in adjuvant formulation (Fig. 1A). In addition, the AddaVax adjuvant alone in PBS was also injected using the same schedule and volume as the negative control group. Sera were collected by tail bleeding according to the regimen illustrated in Fig. 1A. On days 7 and 14 after priming and days 3 and 7 after the second boosting immunization, mice were sacrificed, and sera, spleens, and draining lymph nodes (dLNs) from axilla and inguina were collected.

### Detection of Serum IgG and Specific Antibodies

Serum IgG levels were measured using a Mouse IgG Total ELISA Ready-SET-Go Kit (eBioscience, San Diego, CA, USA) following the manufacturer’s instructions and as described previously (Yu *et al.*^2016^).

Specific antibodies in the serum samples were analyzed using ELISA, as described previously (Yu *et al.*^2016^). To detect antigen-specific antibodies, 96-well plates (JET BIOFIL, Guangzhou, China) were coated at 4 °C overnight with HIV-1 Bal gp120, 06044 gp120T, or HBsAg proteins at a final concentration of 0.5 μg/mL in coating buffer (0.05 mol/L Na_2_CO_3_, 0.05 mol/L NaHCO_3_, pH 9.6). HIV-1 Bal gp120 proteins were obtained through the NIH AIDS Reagent Program, Division of AIDS, NIAID, NIH. After blocking with PBS containing 0.5% Tween-20 and 1% bovine serum albumin, ten fold serially diluted sera were added to each well and incubated at 37 °C for 2 h. The plates were then washed and incubated with horseradish peroxidase-labeled goat anti-mouse IgG (ZSGB-BIO) at a dilution of 1:1000 for 1 h at 37 °C. After washing, the substrates were added, and the reactions were stopped with 2 mol/L H_2_SO_4_. The absorbance at 450 nm (OD_450_) was measured using a Model 550 Microplate Reader (Bio-Rad, Hercules, CA, USA). The endpoint antibody titer in mouse serum was calculated as the log value of the serum dilution with an absorbance value more than two times that of background control wells.

### B-cell Enzyme-Linked Immunospot (ELISpot) Assay

The B-cell ELISpot assay was performed using an ELISpot^PLUS^ for Mouse IgG kit (Mabtech, Nacka Strand, Sweden) as described previously (Yu *et al.*^2016^). Briefly, 96-well MultiScreen-IP filter plates were coated with 10 μg/mL polyclonal goat anti-mouse IgG. After blocking, appropriate numbers of splenocytes were added and incubated at 37 °C for 16–24 h. Then, 100 μL/well of biotin-anti-mouse IgG were added and incubated at room temperature for 2 h. Incubation with alkaline phosphatase-conjugated streptavidin, the substrate reaction, and the stop solution, were performed per the manufacturer’s instructions. Spots representing the total IgG antibody secreting cells (ASCs) were counted using the ChampSpot III ELISpot Analysis System (Beijing, China).

### Flow Cytometry

Single-cell suspensions of dLNs in ice-cold flow cytometry buffer (2% fetal calf serum and 2% mouse serum in PBS) were prepared. To determine the T cells subsets, single cells were stained using the following antibody set: anti-CD3-Pacific Blue, anti-CD4- peridinin-chlorophyll-protein/Cy5.5, anti-PD-1-fluorescein isothiocyanate (FITC), anti-inducible costimulatory molecule (ICOS)-phycoerythrin (PE), anti-CD44-PE/CF594, anti-CD62L-Brilliant Violet 605, anti-C-X-C chemokine receptor 5 (CXCR5), anti-rat-biotin, and streptavidin-PE-Cy7. To determine the B-cell subsets, anti-Fas-PE/CF594, anti-GL-7-FITC, anti-CD19-PE/Cy7, anti-CD69-PE, anti-I-Ab-allophycocyanin (APC), anti-IgM-PE/Cy7, anti-CD138-APC, anti-B220-APC/Cy7, and anti-IgG1-FITC were used. Anti-CXCR5-biotin and streptavidin-PE were purchased from BD Biosciences (Franklin Lakes, NJ, USA). The other fluorescence-labeled antibodies were purchased from BioLegend (San Diego, CA, USA). All isotype-matched control antibodies were purchased from BD Biosciences or BioLegend accordingly. Data were acquired on an LSRFortessa cell analyzer (BD Biosciences) and analyzed using FlowJo software (Tree Star Inc., Ashland, OR, USA). The isotype control settings and gating strategies for T and B subsets are shown in Supplementary Figs. S1 and S2.

### Statistical Analysis

All statistical analyses were conducted using Prism 5 software (GraphPad Software). Differences between the means of two continuous variables were evaluated by the Student’s *t* test with a two-tailed 95% confidence interval. Results with *P* values of less than 0.05 were considered significant.

## Results

### Increased gp120 Immunization Dose Reduced the Requirement for Booster Immunizations without Affecting the Slow Recall Pattern

Previously, we found that specific antibodies were extremely unobvious, even after three Env immunizations (molar ratio of gp120 to HBsAg = 1:1) (Yu *et al.*^2016^). Because the humoral immune response is enhanced as the protein antigen dose increases (Civin *et al.*^1976^; Rweyemamu *et al.*^1984^; Arps *et al.*^1998^), in the present study, we added gp120 protein at 15 μg/mouse (molar ratio of gp120 to HBsAg = 1.5:1). We detected specific antibody responses induced by the first boost with both gp120 (endpoint titer = 2.997 ± 0.095) and HBsAg (endpoint titer = 3.652 ± 0.175; Fig. 1B). However, increases in antibody titers in the gp120 group were subtle compared with those in the HBsAg group. Similarly, after the second boost, the increase in antibody titer induced by gp120 on day 3 (from 3.002 ± 0.097 to 3.021 ± 0.145, *P* = 0.9110) was lower than the increase induced by HBsAg (from 3.584 ± 0.178 to 3.984 ± 0.240, *P* = 0.0433; Fig. 1B). Furthermore, antibody titers to HBsAg from day 3 (day 45) to day 7 (day 49; from 3.984 ± 0.240 to 4.084 ± 0.131, *P* = 0.7287) after the second boost were comparable, whereas that to gp120 (from 3.021 ± 0.145 to 3.640 ± 0.110, *P* = 0.0144) were continuously increased (Fig. 1B). The antibody response patterns to the homologous 06044 gp120T and Bal gp120 were similar (Fig. 1B). The soluble trimeric HIV-1 gp120 elicited significantly slower antibody recall responses than HBsAg, consistent with our previous result, although a relatively large quantity of gp120 was used, implying that increased gp120 immunization dose lessened the requirement for booster immunizations without affecting the slow recall pattern.

### Expansive Nonspecific IgG Responses Induced by gp120 Immunization Occurred in the Primary Immune Response

Because gp120-specific IgG accounted for a very low frequency of serum total IgG, as described in some studies (Fouda *et al.*^2011^; Yu *et al.*^2016^), we defined the total IgG as the amount of nonspecific antibodies. In our previous study, we found that HIV-1 gp120 induced higher levels of nonspecific IgG after repeated boost immunizations (Yu *et al.*^2016^). In the present study, we found that gp120 still elicited higher serum total IgG than did HBsAg, not only after repeated boosts but also immediately after prime immunization (Fig. 1C). Additionally, gp120 also produced more total IgG ASCs than HBsAg after the second boost (Fig. 1D). The above results indicated that HIV-1 gp120 elicited significantly more nonspecific IgG than HBsAg arising from prime immunization.

### Comparable Activation of B Cells But Higher Expression of Major Histocompatibility Complex (MHC) II, Reduced Levels of GC B Cells, and Diminished B Memory Responses Induced by gp120 after Prime Immunization

**Fig. 2:**
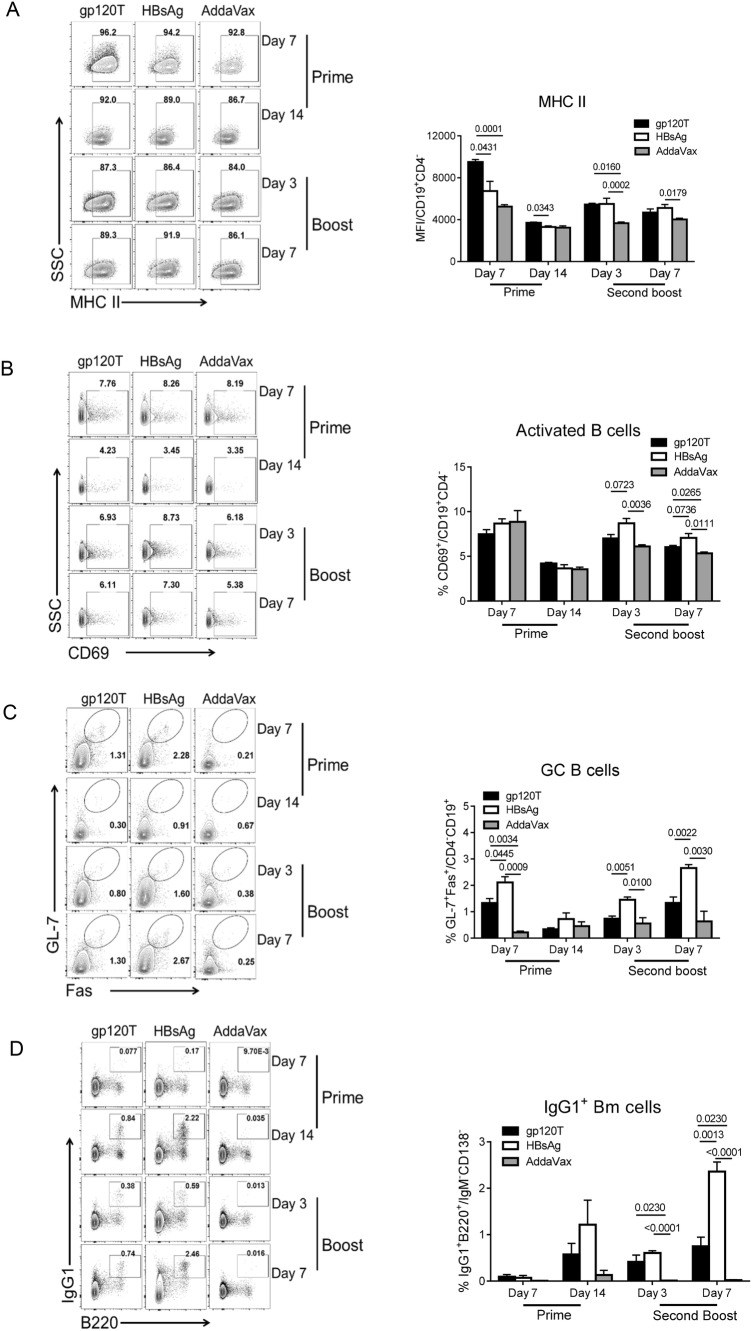
B-cell repertoire induced by prime and boost immunizations with gp120 and HBsAg (n = 4 per group). Mice were immunized with wild-type trimeric gp120 (gp120T) and HBsAg, as described in Fig. 1A, and sacrificed on days 7 and 14 after prime immunization and days 3 and 7 after the second boost. The frequencies of different B cell subsets in the dLNs were analyzed by flow cytometry. Contour plots and statistical graphs of MHC II expression on B cells (**A**), activated B cells (CD69^+^CD19^+^CD4^−^) (**B**), GC B cells (Fas^+^GL-7^+^CD19^+^CD4^−^) (**C**), and IgG1^+^ Bm cells (IgG1^+^B220^+^IgM^−^CD138^−^) (**D**) are shown. Data from a single representative experiment of two independent experiments are shown as means ± standard errors of the means.

Next, we measured the expression of MHC II molecules on B cells, representing the antigen presentation of B cells to the cognate TCR. We found that gp120 elicited more MHC II expression on B cells than HBsAg after priming, but there were no discernible differences after boosting (Fig. 2A). There was transiently higher expression of MHC II on B cells on day 7 after prime immunization, although the expression tapered off by day 14. However, the activated B-cell responses, as measured by CD69 molecule expression, did not differ significantly between the two antigens after prime immunization (Fig. 2B).

More importantly, the GC B-cell proportion in gp120-immunized mice was significantly lower than that in HBsAg-immunized mice after priming, with the same difference after the final boost (Fig. 2C). Finally, we observed elevated proportions of IgG1^+^ Bm cells in gp120-immunized mice on day 14 after prime immunization, although this difference was not statistically significant (Fig. 2D). The final results demonstrated that HBsAg elicited a significantly higher proportion of Bm cells than gp120 on day 7 after the final boost immunization (Fig. 2D). Poor primary GC B cell and Bm cell responses in gp120-immunized mice were consistent with the final slower antibody recall responses.

### Gp120 Induced More PD-1^+^ T Cells and Tfh Cells after Prime Immunization

**Fig. 3:**
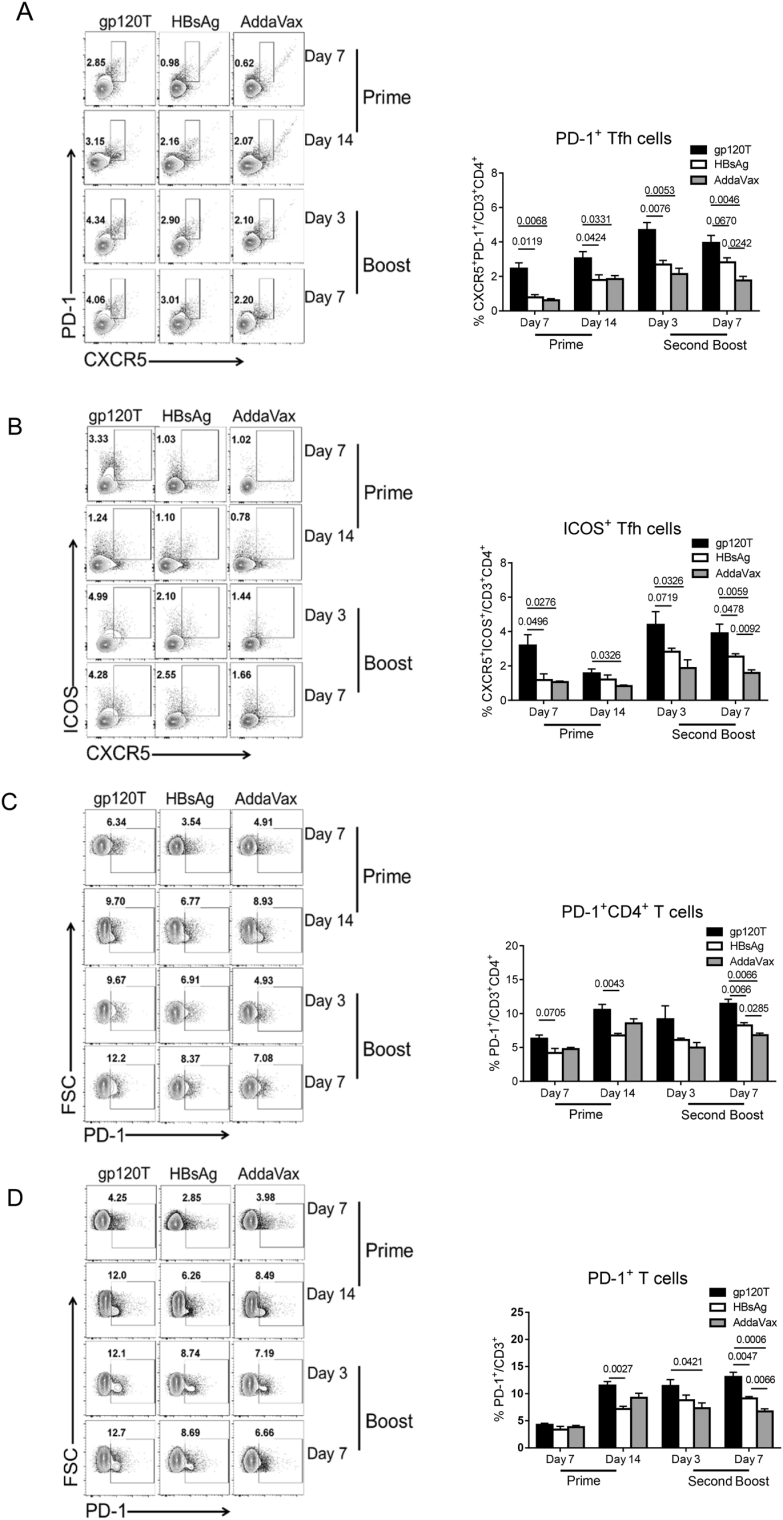

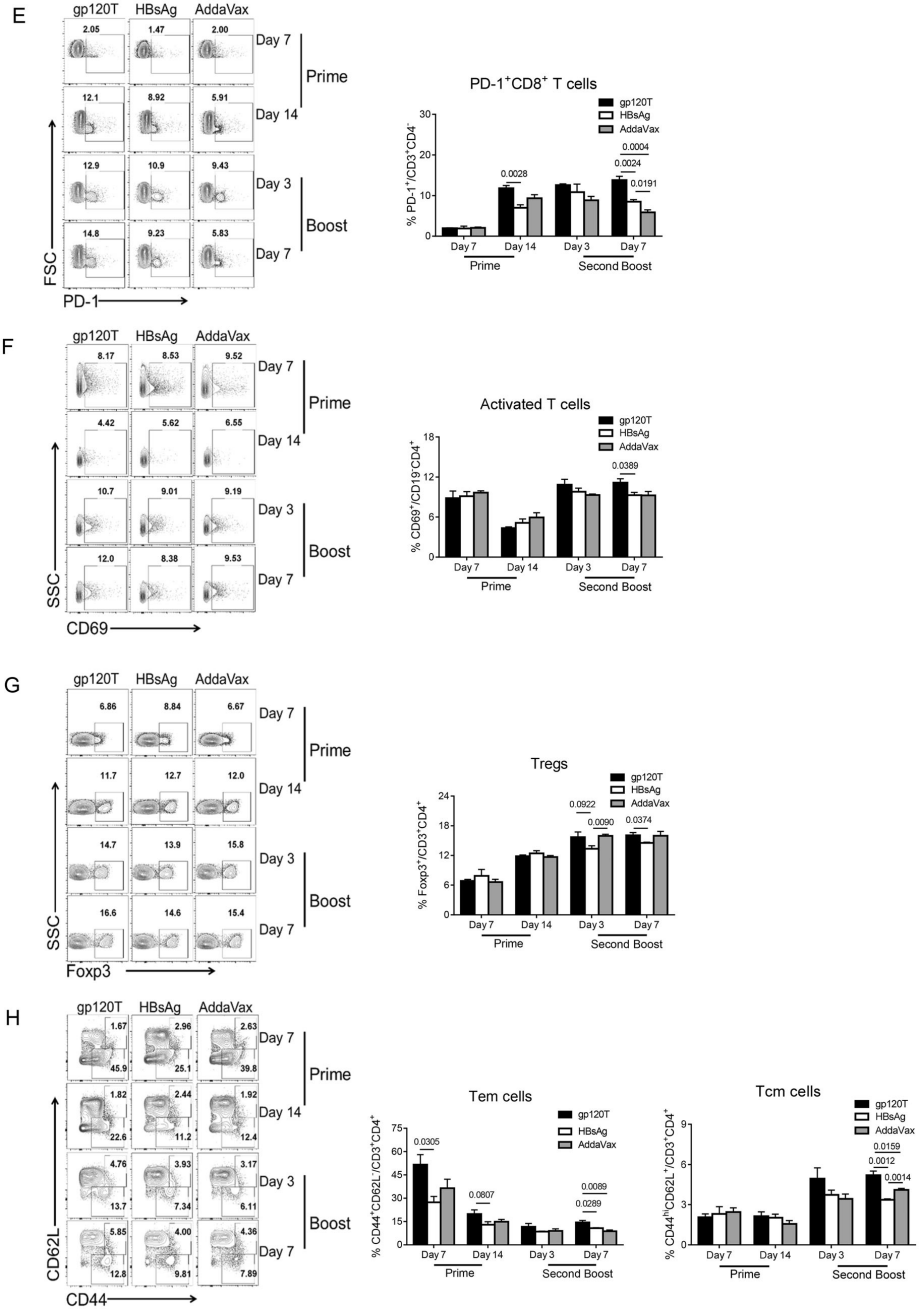
T-cell repertoire induced by prime and boost immunizations with gp120 and HBsAg (n = 4 per group). Mice were immunized with wild-type trimeric gp120 (gp120T) and HBsAg, as described in Fig. 1A, and sacrificed on days 7 and 14 after prime immunization or on days 3 and 7 after the second boost. The frequencies of different T-cell subsets in the dLNs were analyzed by flow cytometry. Contour plots and statistical graphs of PD-1^+^ Tfh cell (PD-1^+^CXCR5^+^CD3^+^CD4^+^) (**A**), ICOS^+^ Tfh cells (ICOS^+^CXCR5^+^CD3^+^CD4^+^) (**B**), PD-1^+^CD3^+^CD4^+^ T cells (**C**), PD-1^+^CD3^+^ T cells (**D**), PD-1^+^CD3^+^CD8^+^ T cells (**E**), activated T cells (CD69^+^CD19^−^CD4^+^) (**F**), Tregs (Foxp3^+^CD3^+^CD4^+^) (**G**), Tem cells (CD44^+^CD62L^−^CD3^+^CD4^+^), and Tcm cells (CD44^hi^CD62L^+^CD3^+^CD4^+^) (**H**) are shown. Data from a single representative experiment of two independent experiments are shown as means ± standard errors of the means.

**Fig. 4:**
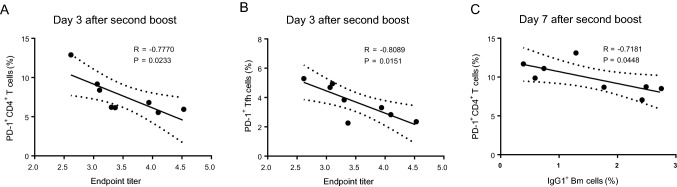
Correlation analyses between proportions of PD-1^+^CD4^+^ T cells or PD-1^+^ Tfh cells and the level of specific antibody responses or IgG1^+^ Bm cells after the second boost immunization. Correlation analysis between the levels of specific antibodies and the proportions of PD-1^+^CD4^+^ T cells (**A**) or PD-1^+^ Tfh cells (**B**) on day 3 after the second boost immunization and correlations between the proportions of PD-1^+^CD4^+^ T cells and IgG1^+^ Bm cells (**C**) on day 7 after the second boost immunization in gp120- and HBsAg-immunized mice.

The effective interaction of Tfh and cognate B cells within germinal centers (GCs) is critical for optimal development of the B-cell repertoire, including Bm cell and antibody responses. However, Tfh cell subsets express CXCR5, the inhibitory receptor PD-1, and the inducible costimulator ICOS. These latter two molecules may divide Tfh cells into functionally different subsets (Eivazi *et al.*^2016^; Jogdand *et al.*^2016^). We have found that gp120 can induce more PD-1^+^CD4^+^ T cells as well as Tfh cells by repeated boosts (Yu *et al.*^2016^). In the current study, we analyzed Tfh cell subsets (CD3^+^CD4^+^CXCR5^+^PD-1^+^ or CD3^+^CD4^+^CXCR5^+^ICOS^+^) after gp120 or HBsAg prime and boost immunizations. We found that after prime immunization, the frequency of PD-1^+^ Tfh, ICOS^+^ Tfh, and PD-1^+^CD4^+^ T cells in gp120-immunized mice was significantly higher than that in HBsAg-immunized mice (Fig. 3A–Fig. 3C). Similar differences were observed on days 3 and 7 following the final boost (Fig. 3A–Fig. 3C), which was consistent with previous results (Yu *et al.*^2016^). Moreover, a similar phenomenon was observed for PD-1^+^CD3^+^ and PD-1^+^CD8^+^ T cells (Fig. 3D, Fig. 3E). The frequency of PD-1^+^ T cells increased slightly after the final boost when compared with prime immunization (Fig. 3C, Fig. 3D, Fig. 3E). Synchronously, the proportions of PD-1^+^ T or PD-1^+^ Tfh cells were negatively correlated with the level of specific antibody responses on day 3 after the second boost immunization (Fig. 4A, 4B), and the proportion of PD-1^+^CD4^+^ cells was negatively correlated with IgG1^+^ Bm cells on day 7 after the second boost (Fig. 4C). Furthermore, the proportions of activated T cells and regulatory T cells (Tregs) in gp120-immunized mice were higher than those in HBsAg-immunized mice after the final boost immunization, although there were no measurable differences after prime immunization (Fig. 3F, Fig. 3G).

The ability of the immune system to “remember” previous encounters with antigen, beginning with the first encounter with antigens, is a cardinal feature of an effective vaccine. We further analyzed the levels of memory T cells. After prime immunization, gp120 produced more effector memory T (Tem) cells but not central memory T (Tcm) cells than did HBsAg (Fig. 3H). After the final boost immunization, gp120 also induced more Tcm and Tem cells (Fig. 3H). The higher percentage of Tem cells after prime immunization may be responsible for the robust Tfh cell response upon re-exposure to gp120. Additionally, higher percentages of Tem cells were observed in all immunized animals after prime immunization, and the levels declined after boost immunization, in contrast to Tcm cells, which may indicate that the formation of good central memory requires repeated antigen stimulation.

**Fig. 5:**
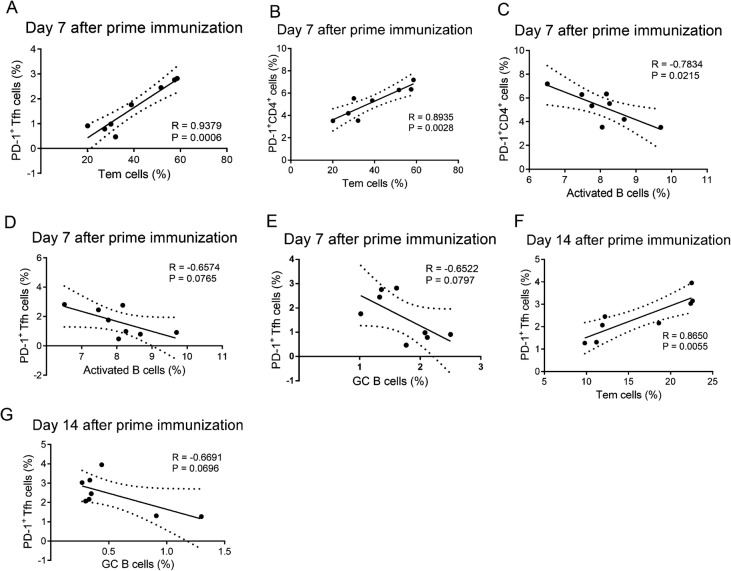
Correlation analyses between the proportions of PD-1^+^CD4^+^ T cells or PD-1^+^ Tfh cells and Tem cells, activated B cells, and GC B cells after prime immunization. Correlation analysis between proportions of PD-1^+^CD4^+^ T cells or PD-1^+^ Tfh cells with Tem cells, activated B cells, or GC B cells on day 7 after prime immunization (**A**–**E**) and the correlations between proportions of PD-1^+^ Tfh cells with Tem cells or GC B cells on day 14 after prime immunization (**F**, **G**) in gp120- and HBsAg-immunized mice.

Notably, on day 7 after prime immunization, we found that lower proportions of PD-1^+^ T and PD-1^+^ Tfh cells were positively correlated with lower proportions of Tem cells and higher proportions of active B cells and (Fig. 5A–5D), whereas the proportions of GC B cells were negatively correlated with PD-1^+^ Tfh cells, although this latter relationship was not significant (Fig. 5E). The correlations between PD-1^+^ Tfh cells and Tem or GC B cells on day 14 after prime immunization were similar to the correlations on day 7 after prime immunization (Fig. 5F, 5G).

## Discussion

Once a vaccine formulation is injected, its initial influence on the magnitude, type, and quality of the downstream immune response, including the generation of immune memory, occurs at the priming event (Ciabattini *et al.*^2016^). However, to date, only a few publications have focused on the initial influence of an immunogen on immune memory responses. A comparison study confirmed that gp120 DNA prime immunization leads to increased Tfh cell differentiation, GC B cells, and specific antibody titers compared with prime immunization with gp120 protein (Hollister *et al.*^2014^). DNA prime immunization also leads to more activated CD4^+^ T cells differentiating into Tfh cells and more GC B cells differentiating into memory cells, indicating the importance of the primary immune response for immunological memory formation (Hollister *et al.*^2014^).

In this study, we compared the antibody elicitation, B-cell differentiation, and Tfh response after prime and boost immunizations with HIV-1 Env gp120 and HBsAg proteins. Compared with our previous study, we found that increasing the amount of Env antigen reduced boost frequency, but did not accelerate antibody recall responses. The development of bNAbs is known to take a long time (over 2 years) in HIV-1-infected patients (Sather *et al.*^2009^) and rarely occurs, even after repeated HIV-1 Env vaccination (Rerks-Ngarm *et al.*^2009^; Sanders *et al.*^2015^; Klasse *et al.*^2016^), which highlights the importance of appropriate responses of memory B cells. Several studies (Evans *et al.*^2004^; Moody *et al.*^2012^; Rouers *et al.*^2017^) have shown that the presence of the antibody recall response in HIV-1-infected patients or clinical vaccine trials is essential for the longevity of protection from infection and generation of NAbs; however, no reports have described the response rates of the antibody recall response to HIV-1. In the current study, compared with HBsAg, a successful vaccine with good recall response, gp120 vaccination elicited a slower antibody recall response, suggesting that the slower antibody response may be an obstacle for effective vaccination against HIV. We also demonstrated that after prime immunization, gp120 induced expansion of nonspecific antibodies and a transient burst of MHC II expression on B cells, but with overall fewer GC B cells than HBsAg, with a similar trend of eliciting a slower antibody recall response as observed in previous results. Generally, naïve B cells are activated by B-cell receptor (BCR)-recognizing antigens and then present peptide antigens to cognate T cells through MHC II. The more antigens B cell surface MHC II molecules present to TCRs on activated T cells, the more help antigen-primed B cells can receive from T cells and the more activated B cells can differentiate into plasma cells and Bm cells (Lanzavecchia 1985; Gitlin *et al.*^2014^). In contrast, in this study, our findings indicated that gp120 may promote more MHC II expression on B cells, but that this antigen may not efficiently promote B-cell proliferation in GCs and IgG1^+^ Bm cell differentiation after prime immunization. More MHC II on B cells did not lead to the development of preferable immune responses in gp120-immunized mice.

For antigen presentation in prime immunization, the antibody response is initiated when naïve B cells bind to foreign antigens via its surface immunoglobulin, namely the BCR. The BCR-antigen macromolecular cluster forms and contracts into a mature immunological synapse, followed by internalization of the antigen (Spillane and Tolar 2017). Small and soluble antigens (less than 70 kDa) can quickly reach the follicle and are acquired by follicular B cells (Roozendaal *et al.*^2009^), whereas larger antigens, such as viruses or immune complexes are retained within the subcapsular sinus and displayed by macrophages and follicular dendritic cells (Carrasco and Batista 2007; Junt *et al.*^2007^; Phan *et al.*^2007^). The trimeric gp120 used for the present study displayed only three gp120 protomers on each trimer molecule. Given the large size of trimeric gp120, it is difficult for it to be recognized by the BCR directly. Rather, it requires presentation from several types of cells. Morever, HIV Env proteins are not recognized by the germline BCR of bNAbs, leading to extremely low binding affinity between Env and BCR on naïve B cells, which may not be sufficient for the promotion of antigen-specific B-cell formation (Jardine *et al.*^2013^; Liao *et al.*^2013^). In contrast, in our previous study, native-PAGE showed that the molecular weight of HBsAg is over 1024 kDa, although the actual molecular weight of a single HBsAg molecule is about 23 kDa, suggesting that HBsAg molecules were highly aggregated and may form into particles (Yu *et al.*^2016^). Moreover, electron microscopy analysis has demonstrated that the HBsAg immunogen is highly aggregated and self-assembles into small nanoparticles (Zhao *et al.*^2013^; Yu *et al.*^2016^). Nanoparticles display abundant repeated epitopes that can crosslink with the BCR, which improves B-cell activation, leading to heightened affinity maturation and the development of long-lived plasma cells (Sliepen and Sanders 2016).

The PD-1/PD-L1 pathway negatively regulates the humoral response (Good-Jacobson *et al.*^2010^). Additionally, PD-1 blockade may increase the proliferation of Bm cells and the production of simian immunodeficiency virus (SIV) Env-specific antibodies (Velu *et al.*^2009^). In the current study, gp120 exposure induces higher proportions of PD-1^+^ T cells and PD-1- or ICOS-expressing Tfh cells. Nonetheless, gp120 exposure also led to poor B cell repertoire development, including reduced numbers of Bm cells and poor antibody recall responses. We found that a higher proportion of Bm cells were positively correlated with lower proportions of PD-1^+^CD4^+^ cells. Furthermore, higher proportions of PD-1^+^CD4^+^ T cells and PD-1^+^ Tfh cells were induced by gp120 prime immunization. Therefore, PD-1 could be defined as an Env-induced inhibitory receptor that can attenuate and/or delay recall antibody responses by adversely affecting Tfh cell/B cell interaction. Cubas *et al.* (2013) have proven that, despite the expansion of Tfh cells in HIV-1-infected individuals, the cells are unable to provide adequate help to B cells due to the engagement of PD-1 on Tfh cells, leading to reduction in cell proliferation, activation, ICOS expression, and IL-21 secretion. Good-Jacobson *et al.* (2010) have shown that in PD-L- or PD-1-deficient mice, increased GC B-cell death corresponded to quantitative defects in PC numbers; however, the remaining PCs were of higher affinity than wild-type cells (Good-Jacobson *et al.*^2010^). Thus, inhibition of PD-1/PD-L1 signaling before or during prime immunization is a potential strategy to improve final slow antibody recall response and poor immune memory response. Unfortunately, we did not examine whether there were similar differences in the expression patterns of PD-L1 and PD-L2 on B cells. Extensive profiling of the characteristics of immune responses induced by HIV-1 gp120 and HBsAg prime immunization may be essential for the rational design of vaccination strategies.

In addition to the structural differences between gp120 and HBsAg, differences in glycosylation patterns may also lead to differences in humoral immune responses. Indeed, Zeng *et al.* (2014) demonstrated that carbohydrate antigens not only initiate specific antibody responses with help from the innate immune system but also activate the T cell-independent pathway. However, the persistence of B cell responses is poor in the absence of CD4^+^ T-cell responses (Bergmann-Leitner and Leitner 2014). Indeed, during HIV or SIV infection, with the loss of CD4^+^ T cells and disease progression to acquired immunodeficiency syndrome, Env-specific antibodies remain surprisingly high, indicating that at least some of these are T-cell independent (Zwart *et al.*^1994^; Binley *et al.*^1997^; He *et al.*^2006^). Furthermore, membrane-bound trimeric Env immunization in immunocompetent and T cell-deficient mice also demonstrated that the T cell-independent secondary antibody response of Env protein may prevent T cell-dependent affinity maturation (Nabi *et al.*^2012^). Furthermore, optimal T-cell activation requires signals delivered via TCR engagement of the MHC/antigen complex (Carter and Carreno 2003). However, glycosylation of the antigen can affect cellular uptake, proteolytic processing, and presentation by MHC II (Wolfert and Boons 2013). We hypothesized that the carbohydrate moieties may be involved in the poor recall responses and stronger nonspecific antibody responses.

Unlike the HBsAg vaccine, which forms virus-like particles and has only very few glycans, most recombinant HIV-1 Env proteins tested in immunogenicity studies to date do not assemble into higher order structures and are heavily glycosylated. These two properties may affect the humoral response.

According to our findings, we deduced that even though HBsAg did not induce more Tfh cells, a lower frequency of PD-1^+^ T cells was obtained, and a better GC reaction and Bm cell response were observed after prime immunization compared with the responses to HIV-1 gp120. This was the major contributor to the final different patterns of recall antibody responses elicited by the two immunogens. The above results seemed to suggest that the formation of appropriate primary immune responses after HIV-1 Env prime immunization had critical effects on the final antibody recall and immune memory responses. Hence, Env immunogens should be designed to display repeated conformational epitopes to minimize PD-1 expression and strengthen the GC B cell and Bm cell responses as prime antigens. In contrast, strategies to lessen the PD-1/PD-L signaling pathway may potentially be utilized during the primary immune phase to improve HIV-1 Env-induced immune responses. However, one limitation of this study was that we did not trace antigen delivery* in vivo* after prime immunization. Additionally, we did not examine whether the trimeric gp120 was well recognized by the BCR. Accordingly, further studies are needed to assess these factors.

## Electronic supplementary material

Below is the link to the electronic supplementary material.

Supplementary material 1 (PDF 771 kb)
