# Supplementary material for: Divergent Primary Immune Responses Induced by Human Immunodeficiency Virus-1 gp120 and Hepatitis B Surface Antigen Determine Antibody Recall Responses
Source: Virol Sin. 2018 Dec 19;33(6):502–14. doi: 10.1007/s12250-018-0074-6 (PMC6335216; doi:10.1007/s12250-018-0074-6)
Supplement: Supplementary file 1 — Supplementary material 1 (PDF 771 kb) [file 12250_2018_74_MOESM1_ESM.pdf]

VIROLOGICA SINICA

Electronic Supplementary Material

Divergent Primary Immune Responses Induced by Human Immunodeficiency Virus-1 gp120 and Hepatitis B Surface Antigen Determine Antibody Recall Responses

Li Yuan[1], Wen-Jiang Chen[1], Jia-Ye Wang[1, 2, 3], Yan Li[1, 2, 3], Dan Tian[1], Ming-Xia Wang[1], Hao-Tong Yu[1], Ying-Chu Xu[1], Di Li[1, 2, 3], Min Zhuang[1, 2, 3], Hong Ling[1, 2, 3, 4,,✉]

1. Department of Microbiology, Harbin Medical University, Harbin 150081, China,
2. Heilongjiang Provincial Key Laboratory of Infection and Immunity, Key Laboratory of Pathogen Biology, Harbin 150081, China
3. Wu Lien-Teh Institute, Harbin Medical University, Harbin 150081, China
4. Department of Immunology, Harbin Medical University, Harbin 150081, China

Supporting information to DOI: 10.1007/s12250-018-0074-6

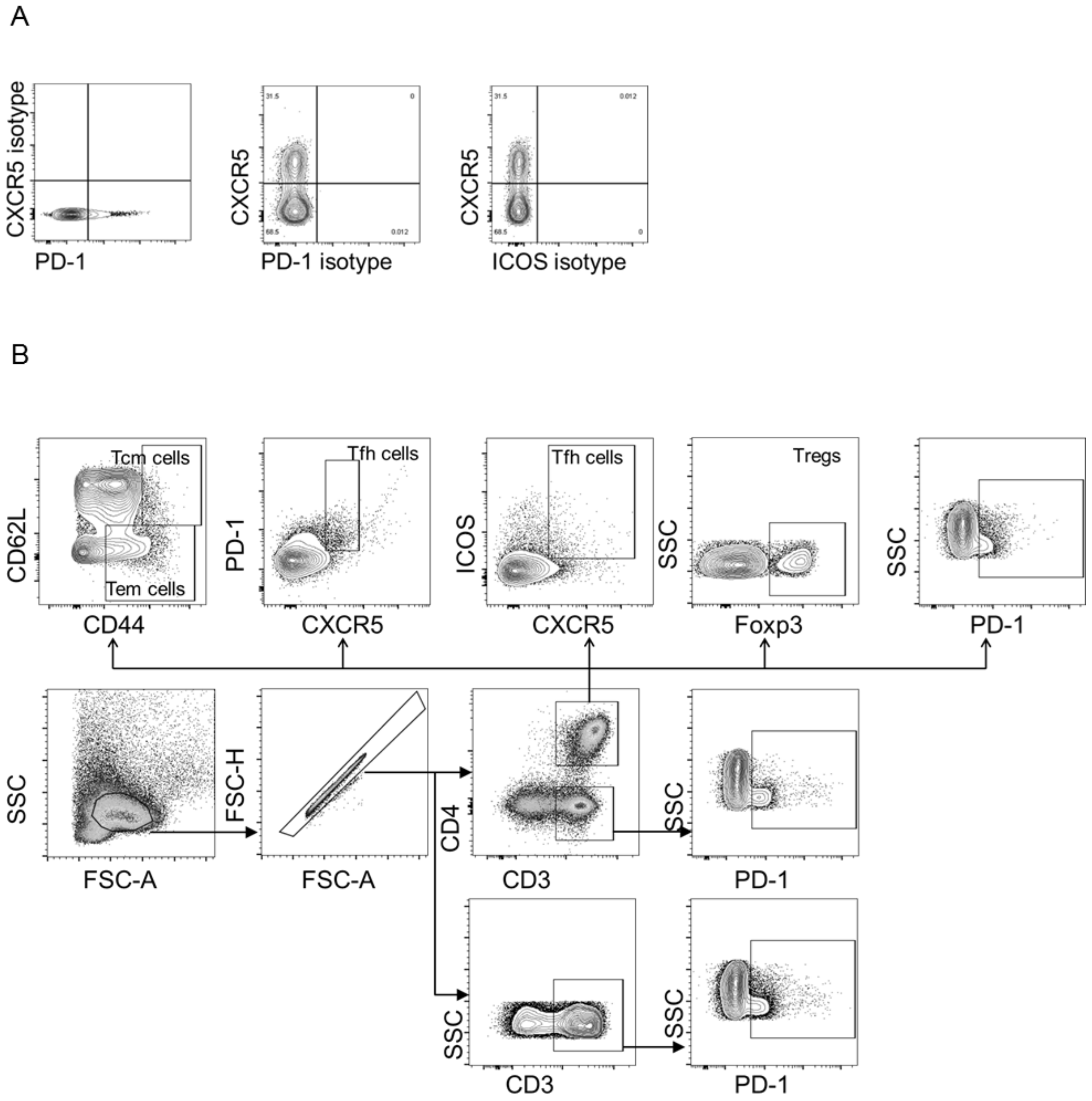

Fig. S1. Isotype control settings and gating strategies for T-cell subsets from mouse dLNs (n = 4 per group). **(A)** Positive and negative boundaries were determined using fluorescence minus one controls of corresponding isotype antibodies. (**B**) PD-1$^+$ Tfh cells, ICOS$^+$ Tfh cells, Tregs, Tem cells, and Tcm cells were gated as PD-1$^+$CXCR5$^+$CD3$^+$CD4$^+$, ICOS$^+$CXCR5$^+$CD3$^+$CD4$^+$, Foxp3$^+$CD3$^+$CD4$^+$, CD44$^+$CD62L$^-$CD3$^+$CD4$^+$, and CD44^{hi}CD62L$^+$CD3$^+$CD4$^+$ cell subsets, respectively.

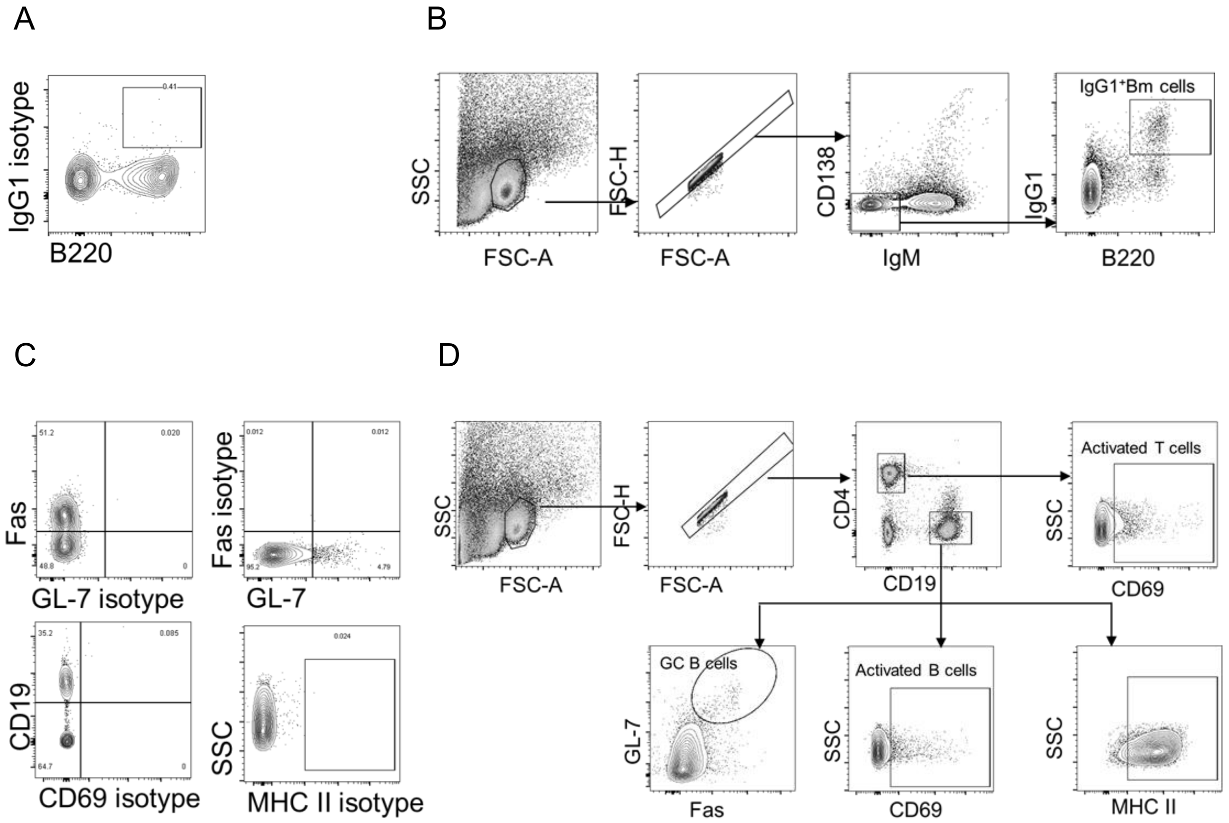

Fig. S2. Isotype control settings and gating strategies for B cell subsets from mouse dLNs (n = 4 per group). (**A, C**) Positive and negative boundaries were determined using fluorescence minus one controls of corresponding isotype antibodies. (**B**) IgG1$^+$ Bm cells were gated as IgG1$^+$B220$^+$IgM$^-$CD138$^-$ cell subsets. (**D**) GC B cells, activated B cells, and activated T cells were gated as Fas$^+$GL-7$^+$CD19$^+$CD4$^-$, CD69$^+$CD19$^+$CD4$^-$, and CD69$^+$CD19$^-$CD4$^+$ cell subsets, respectively.
